# The Distribution Characteristics and Applications for Maternal Cells on Chicken Egg Vitelline Membrane

**DOI:** 10.1038/s41598-017-06996-1

**Published:** 2017-07-26

**Authors:** Quanlin Li, Wenbo Li, Xingzheng Li, Lulu Liu, Ying Zhang, Yuying Guo, Xia Chen, Guiyun Xu, Jiangxia Zheng

**Affiliations:** 1 National Engineering Laboratory for Animal Breeding, MOA Key Laboratory of Animal Genetics and Breeding, College of Animal Science and Technology, China Agricultural University, Beijing, 100193 China; 2 College of Animal Science and Technology, Heilongjiang Bayi Agricultural University, Daqing, 163319 China

## Abstract

The major components of vitelline membrane (VM) are ovomucin, VM outer (VMO) I and VMO II. At present, the distribution pattern of maternal cells on the VM has not been described in detail. In this study, the existence and distribution characteristics of maternal cells on VM were observed. There were more than 3.2 × 10^5^ somatic cells on VM, which were uneven distributed. The calcein AM/PI staining of the maternal cells on the VM showed that the cells’ viability changed with the freshness of the eggs, and that the maternal cells gradually underwent apoptosis and became degraded. The results of morphology of different tissues indicated that the most of maternal cells on the VM were granulosa cells. Moreover, the karyotype of the cultured granulosa cells, which is the main source of cells on VM, were identified as the normal diploid karyotype of chicken. Furthermore, the VM DNA extracted from chickens and quails, which represent the eggs of different size, was adequate for further genetic analysis. The VM DNA was easily accessible and relatively constant, without cross-contamination. Therefore, the VM DNA could potentially be applied for the molecular traceability between eggs and chickens, and be beneficial in avian ecology research studies.

## Introduction

The components of avian egg are, from the inside to the outside, as follows: egg yolk, vitelline membrane (VM), albumen, eggshell membrane, and eggshell. The VM forms the last barrier to microbial infection during embryo development, and plays an important role in fertilization. The structure of the VM consists of three layers: the inner layer is secreted by the granulosa cells surrounding the oocyte in the follicle before ovulation^1, 2^, and the middle and outer layers are both formed in the infundibulum part of the hen’s oviduct. Previous studies have revealed that a hen’s VM contains mainly protein compounds (>80%), along with some carbohydrates and lipids^3, 4^. Kido was able to successfully solubilize VM in sodium dodecyl sulfate (SDS), and the results revealed the presence of three major components (designated GP-I, GP-II, GP-III) which were determined to be glycoprotein at a later date^5, 6^. Along with GP-IV, there are mainly four proteins in the inner layer, and those in the outer layer are ovomucin, lysozyme C, lectin, VM outer (VMO) I, and VMO II^7^.

The traditional protocols of genetic investigations of avian populations requires obtaining the blood and/or tissue samples. The majority of avian studies employ the capturing and handling of the young and parents in order to draw blood for DNA analysis. Sometimes maternal genotyping requires the collection of contour feathers from nests, or the destructive sampling of eggs. In the studies conducted by Egloff, the genomic DNA obtained from within eggshell matrices and their corresponding parents verified the presence of maternal DNA in the eggshell matrix in 100% of the herring gull nests which were assessed. The use of non-destructive sampling methods to collect genetic material from wildlife has allowed researchers to minimize the disturbance^8^. Gregory Schmaltz demonstrated that avian maternal DNA can be isolated in a non-invasive and non-destructive way from the external surfaces of eggs. However, these methods are easily confused by contaminations due to the impurities found in the environments, and the quantities of the DNA were found to be limited and varied^9^. Strausberger indicated that a single egg could provide both maternal and offspring DNA. The genotypes for three or four loci from the embryos and shell samples were also obtained^10^. Arnold sampled the blastoderm/disc, VM, and a mixture of both. The results indicated that the VM was a considerable source of maternal and most likely paternal contamination^11^.

In this study, the maternal cells on the VM were observed, and the potential source of maternal cells on the VM were analyzed in difference tissues. Moreover, the patterns of the cells’ apoptosis with the prolonging of the eggs’ storage periods were also described. Using the DNA extracted from the VM, this study built the molecular traceability between the egg and bird by means of microsatellite genotypes. The method was able to reliably acquire the genetic materials from the smaller bird for the taxonomy, ecology, and evolutionary examinations.

## Results

### General architecture of the VM and maternal cells: TEM

**Figure 1:**
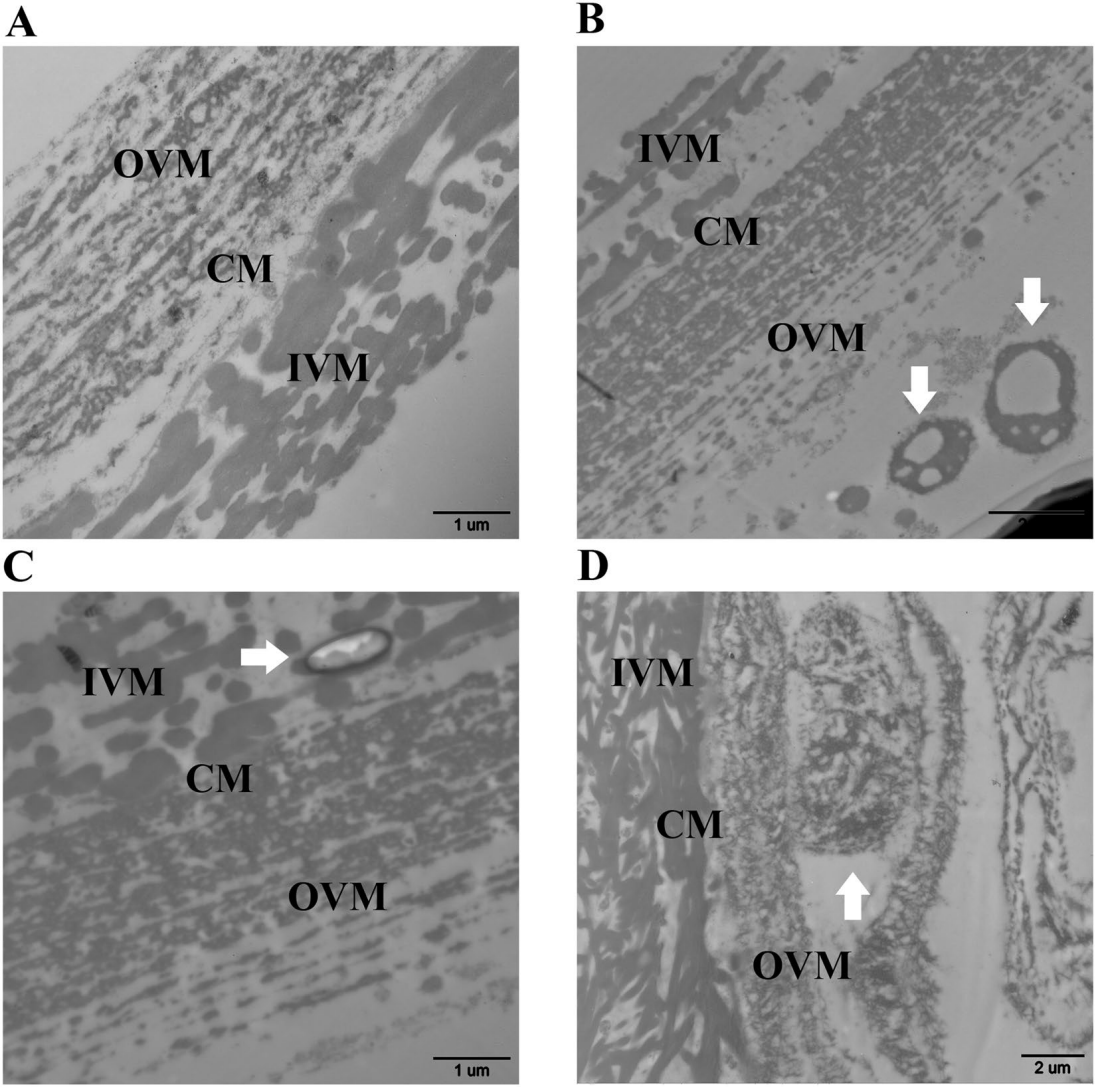
Transmission electron microscopy (TEM) images of the maternal cells on the VM. (**A**) The typical three layers of the VM (×20k). (**B**) Granulosa cells (arrowhead) on the OVM (×12k). (**C**) Elongated nuclei (arrowhead) stuck between the CM and IVM (×20k). (**D**) Apoptotic granulosa cells (arrowhead) on the OVM (×8k). OVM: outer VM; CM: continuous membrane; IVM: inner VM.

In this study, the TEM images showed two structural layers which were separated by continuous membrane (Fig. 1A). The maternal cells with different status could be observed on each layer of the VM (Fig. 1B–D).

### Maternal cells on the VM: LSCM

**Figure 2:**
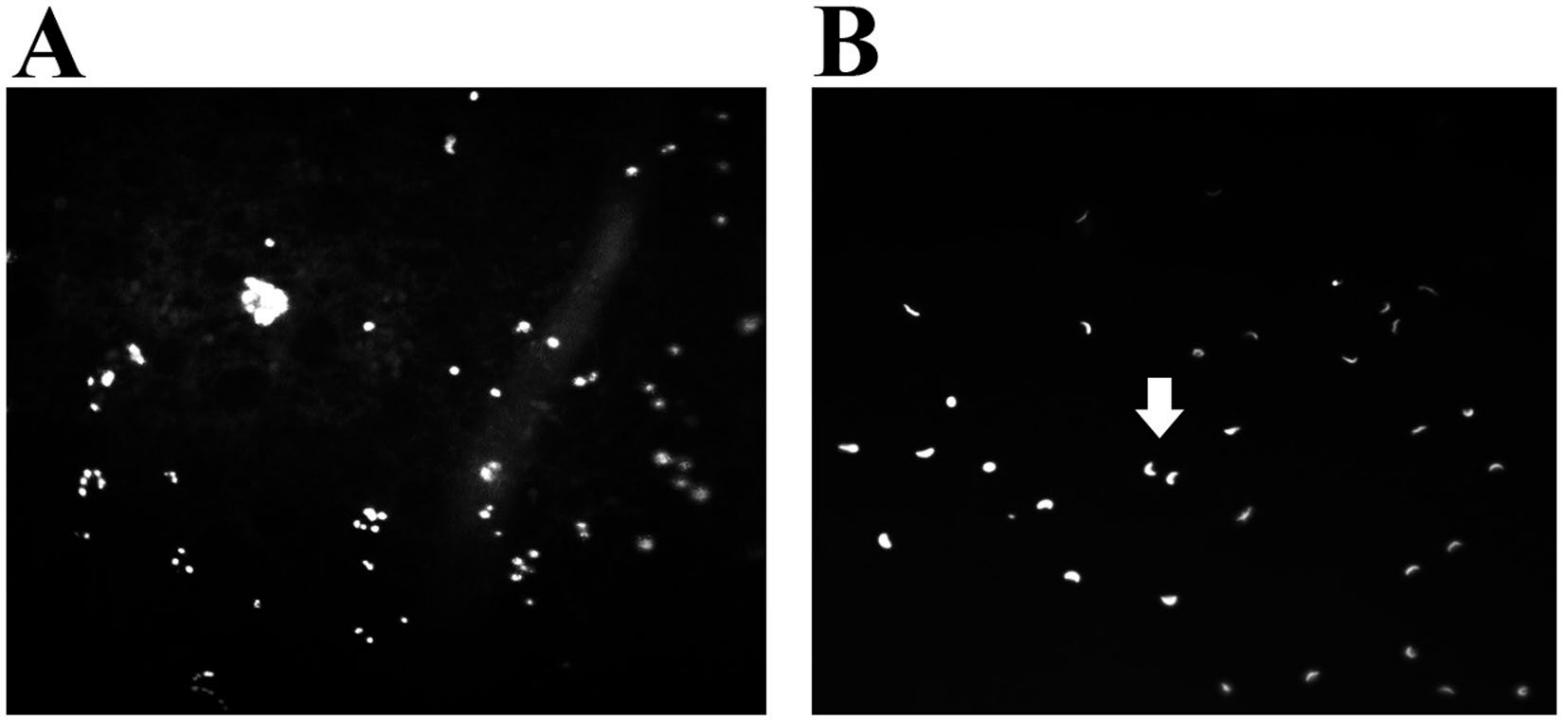
Fluorescent Hoechst 33342 staining of the maternal cells and sperms on the VM. (**A**) The maternal cells on the VM of the unfertilized eggs (×200). (**B**) The sperms on the VM of the fertilized eggs (×200).

A Hoechst 33342 nuclei stain was used to confirm the existent of the maternal cells on the VM. The nuclei of the maternal cells of the unfertilized VM fluoresce were found to be as bright as the nuclei of the sperm of fertilized VM when examined by LSCM (Fig. 2). The possibility of bacterial contamination was excluded by the bacterial gene specific polymerase chain reaction (PCR). The results are not shown here, and are available upon request.

### Egg quality and maternal cell viability at different storage times

**Figure 3:**
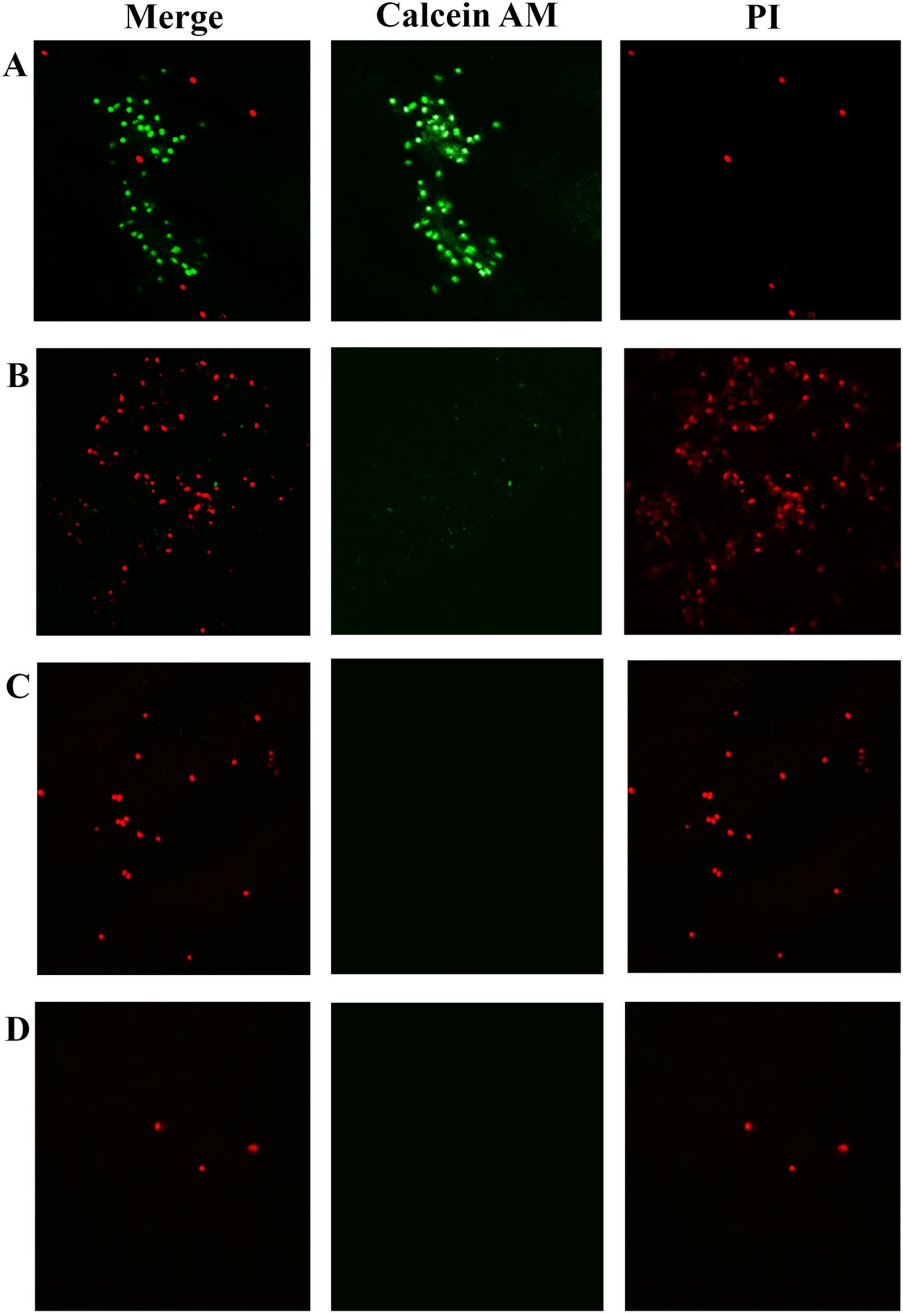
Calcein-AM/PI live/dead staining of the maternal cells on the VM. (**A**) Maternal cells on the VM at the D1 storage time (×200). (**B**) Maternal cells on the VM at the D7 storage time (×200). (**C**) Maternal cells on the VM at the D14 storage time (×200). (**D**) Maternal cells on the VM at the D21 storage time (×200). The live cells were stained green, and the dead cells were stained red.

**Figure 4:**
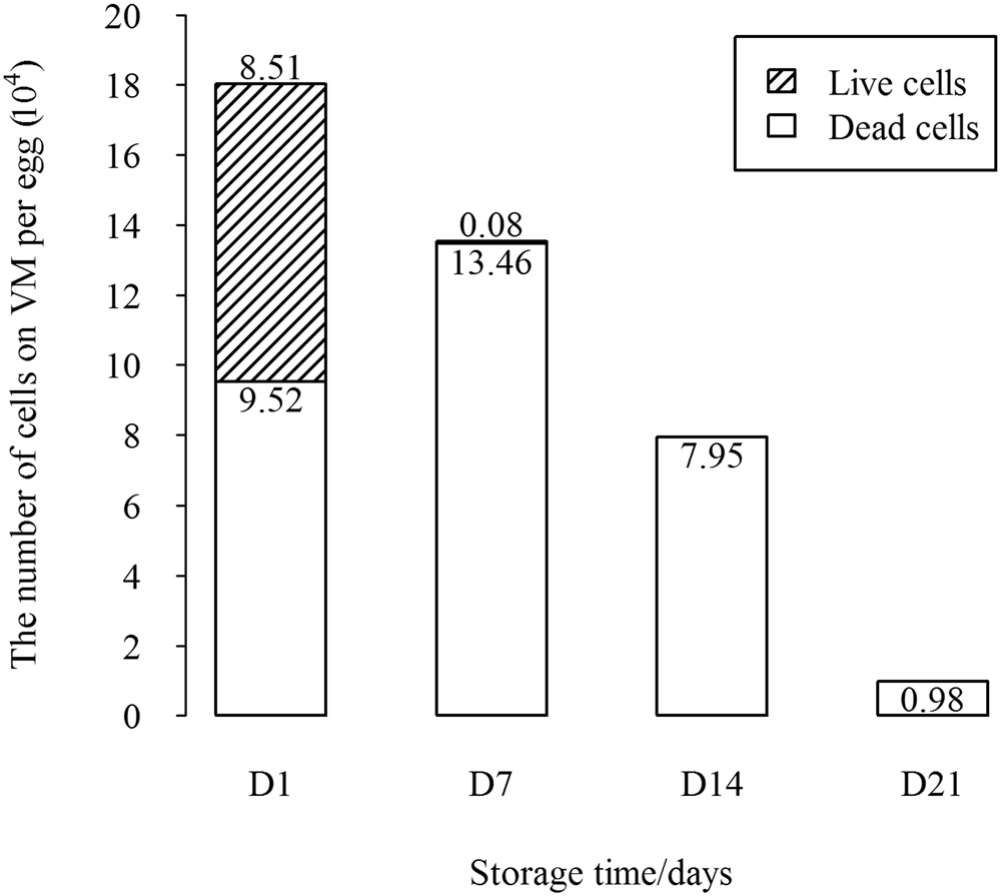
Changes in the live and dead cells of the total cell amount during the storage times of the unfertilized eggs (15 cm^2^).

The DNA concentrations on the VM of chicken and quail eggs decreased significantly with increasing storage time (*P* < 0.05). Similar results were observed on the HU score of chicken eggs. The results of the live/dead staining of the maternal cells on the VM indicated that almost 50% of the cells were viable at the D1 storage time. The cells gradually underwent apoptosis and became degraded with the extensions of storage times (Figs 3 and 4; Table 1). The rate of the live maternal cells decreased dramatically after D7. Also, by D21 there were few cells. The success of the PCR amplification of the VM samples in the microsatellite genotyping also was found to decline with the decreases of the maternal cells as follows: 100% for D1; 84.21% for D7; and 81.58% for D14.

**Table 1:** Results of the egg quality measurements. ^1^Thiry eggs were detected in each group; ^2^The eggs were detected in each group. Note that the 10 eggs used for LSCM were not derived from the 30 eggs used for DNA concentration and Haugh Unit determination. Means ± SD: the same column without a common superscript differ significantly at *P* < 0.05.

Storage time (Days)	Chicken						Quail	DNA concentration (ng/VM)^1^
	Egg weight (g)^1^	DNA concentration (ng/VM)^1^	Haugh Unit^1^	Live cells (0.4 mm^2^)^2^	Dead cells (0.4 mm^2^)^2^	Total cells (0.4 mm^2^)^2^	Egg weight (g)^1^	
1	56.4 ± 4.8^a^	749 ± 240^a^	87.0 ± 5.7^a^	22.70 ± 12.04^a^	25.40 ± 9.35^b^	48.10 ± 9.86^a^	11.2 ± 1.0^a^	530 ± 156^a^
7	54.5 ± 3.9^b^	529 ± 93^b^	68.4 ± 6.4^b^	0.20 ± 0.63^b^	35.90 ± 12.18^a^	36.10 ± 12.12^b^	11.1 ± 1.1^a^	365 ± 59^b^
14	54.8 ± 3.9^b^	535 ± 95^b^	56.8 ± 8.6^c^	0.00 ± 0.00^b^	21.20 ± 5.12^b^	21.20 ± 5.12^c^	11.4 ± 1.1^a^	389 ± 95^b^
21	54.3 ± 3.9^b^	549 ± 329^b^	55.6 ± 8.3^c^	0.00 ± 0.00^b^	2.60 ± 1.43^c^	2.60 ± 1.43^d^	—	—

### Identification of the follicle granulosa cell, the major maternal cell type on VM at different tissues

**Figure 5:**
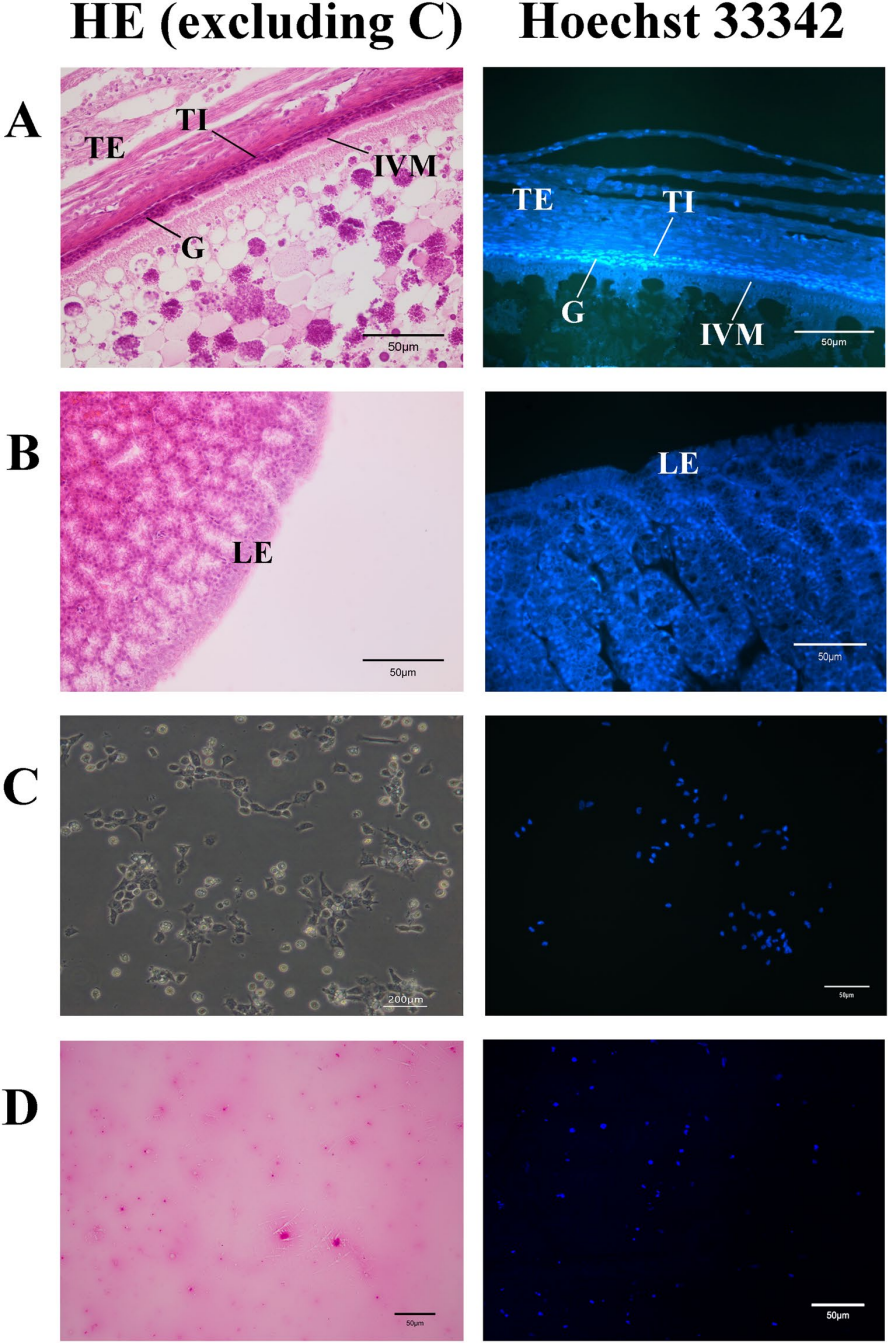
Histology observation of the follicle, infundibulum, granulosa cells and VM using HE and Fluorescent Hoechst 33342 staining. (**A**) The cross-sections of the enveloping layers of SYF (Φ 6–8 mm,×400). TE: theca externa; TI: theca interna; G: granulosa; IVM: inner vitelline membrane. (**B**) The cross-sections of the Infundibulum (×400). LE: luminal epithelium (numerous ciliated cells and very few secretory cells). (**C**) The granulosa cells from SYF after culture for 48 h (×200). The granulosa cells on the left side were directly examined without staining. (**D**) The maternal cell on the VM of 1d unfertilized egg (×200).

The gross morphology of the enveloping layers of SYF is illustrated in Fig. 5A. It was a complex layer of connective tissue and can be divided into the following: a) The theca externa with lightly stained cells. b) The theca interna with densely stained cells. c) The granulosa cells, which were adjacent to the surface of the oocyte, showed the deepest stain signal with both HE and Hoechst33342. d) The inner VM without any stain. Figure 5B shows the cross-sections of the infundibulum with an elaborate folding of mucosa. The luminal epithelium of the mucosa consisted of cilia and very few secreting cells, with less staining by HE and Hoechst33342. The granulosa cells cultured in monolayers displayed the typical features of this cell population. As shown in Fig. 5C, cells plated in culture dishes grew in tight patches of flattened, slightly rounded, epithelioid-like cells. There were the partial apoptotic granulosa cells, which showed changes of apoptosis, including blebbing, cell shrinkage and nuclear fragmentation. The cultured granulosa cells showed strong Hoechst33342 stain signals similar to the granulosa cells layer of SYF. For the staining results on the VM, the partial apoptotic granulosa cells were observed to have uneven distribution (Fig. 5D).

### Karyotype analysis of granulosa cells

**Figure 6:**
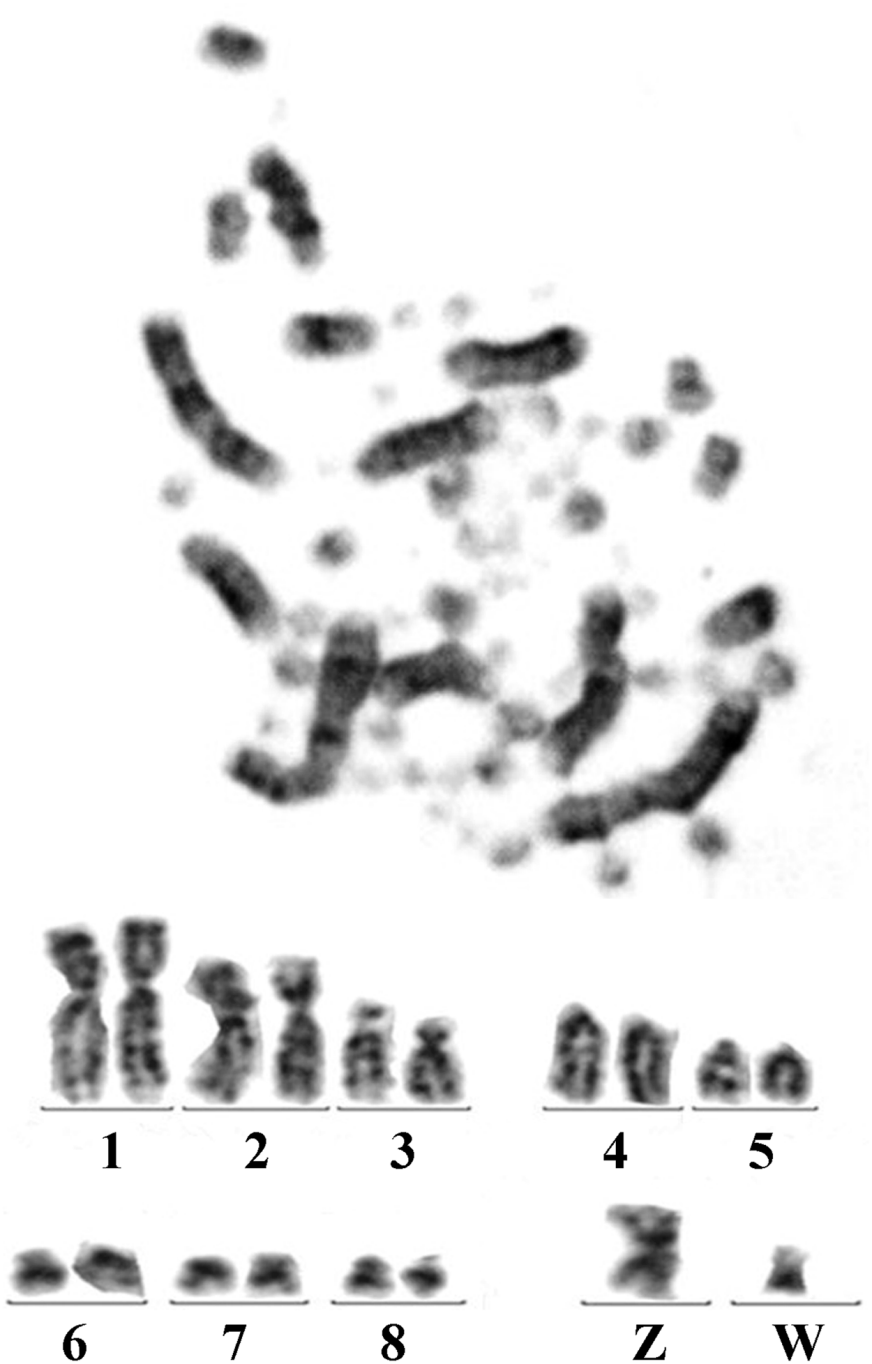
The karyotype of the cultured granulosa cells.

Karyotype analysis was performed based on the observation of metaphase (Fig. 6). There were 39 pairs of chromosomes (2n) in the chicken granulosa cells. Nine pairs of chromosomes, including 8 pairs of larger chromosome and a pair of sex chromosome (ZW), were identified. The other 30 microchromosomes, which may have been telocentric or acrocentric chromosomes, were hard to distinguish.

### Nei standard genetic distances and visualized FCA

**Figure 7:**
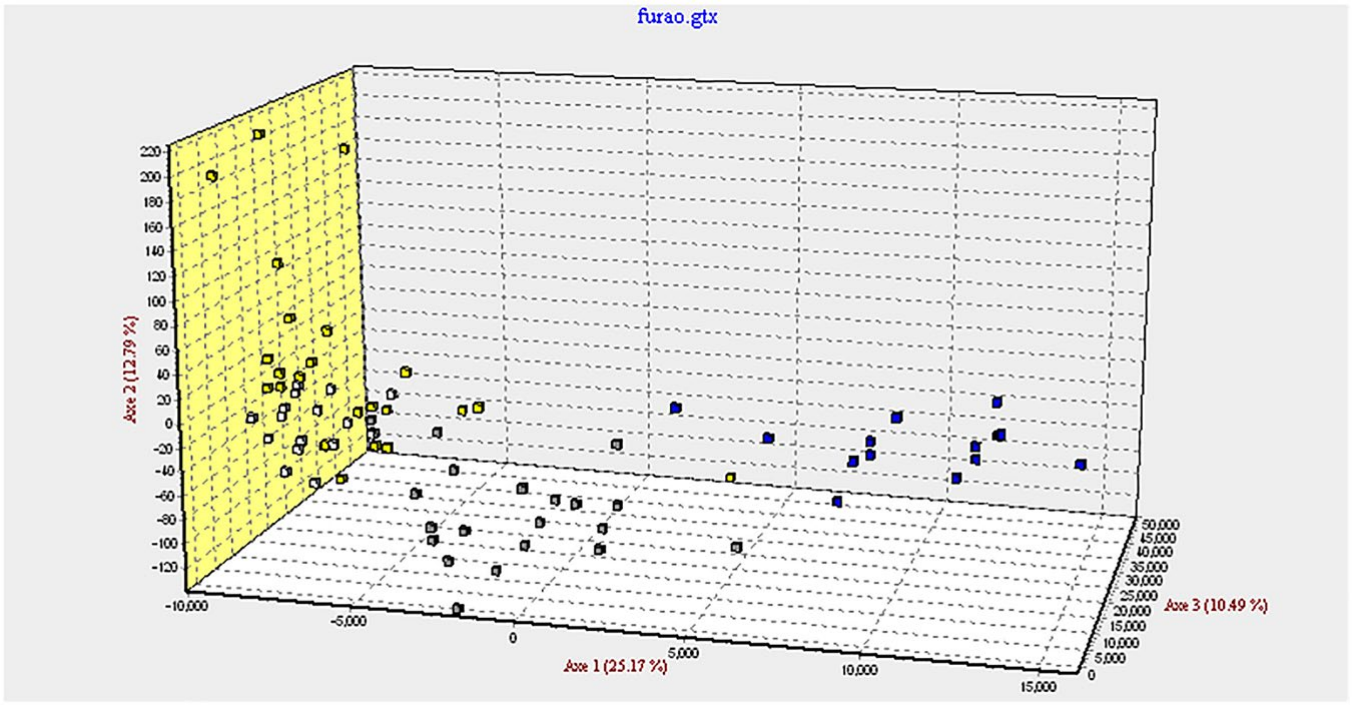
Three-dimensional scatter plots for the individuals of the four groups based on the factor correspondence analysis (FCA). Each axis represents one principal factor: White block: WLA-VM; Yellow block: WLA-B; Gray block: WLB-B; Blue block: RIR-B.

Significant genetic differentiations were found among four chicken groups that produced completely different colored eggs. Also, the WLA-VM and WLA-B showed the closest relationship (Fig. 7; Table 2).

**Table 2:** Matrix of the genetic distances among the four groups. The code letters correspond to the group as follows: WLA-VM represents the VM of the White Leghorn stock A; WLA-B represents the blood of the White Leghorn stock A; WLB-B is the blood of White Leghorn stock B; and RIR-B indicates the blood of Rhode Island Red stock (Fig. 5).

Breed	WLA-VM	WLA-B	WLB-B
WLA-B	0.096		
WLB-B	0.412	0.603	
RIR-B	2.200	1.356	0.755

The FCA analysis also revealed the genetic relationship between the suspected individuals (WLA-VM), and the VM belonging to the reference population (WLA-B), in terms of the allele frequency. The results were in accordance with the Nei standard of genetic distances.

In addition to the microsatellite genotyping, this study also successfully amplified the 5′-end portion of *cox1* gene. It is the standard DNA barcoding region in the mitochondria, and is used to distinguish the related taxa^12–14^. The results are not shown here, and are available upon request.

## Discussion

The typical three layers of the VM, along with the maternal cells on them, were observed in this study using TEM. The maternal cells may have been granulosa cells, and were located at each layer of the VM. Waclawek and Takeuchi found that the inner layer of the VM was secreted by the granulosa cells surrounding the oocyte in the follicle before ovulation, while the middle and outer layers were both formed in the infundibulum part of the hen’s oviduct^1, 2^. Taking the tissue structures of the follicle and infundibulum into account, the layer of granulosa cells in the follicle was proximally adjacent to the inner layer of the VM. The granulosa cells were the most likely cells to be attached to the oocyte when it was released from the follicle in the ovary, according to present and previous studies^12–14^. Madekurozwa previously examined the ultrastructure features of the follicular walls in the developing follicles of sexually immature ostrich^15^. It was observed that the granulosa cells of Madekurozwa’s research were also similar to the present study’s results.

Previous studies have shown that granulosa cells can be identified by the morphology and immunostaining of the FSH receptor (FSHR), as the granulosa cells are the only cell type that express FHSR^16–21^. In order to further determine that the granulosa cell is the major maternal cell type attached to the VM, we attempted to use the immunohistochemial staining of FSHR to identify the VM granulosa cell^22^. However, there is no FSHR antibody specifically react on avian. To supplement the results, we observed the morphology of the granulosa cells at the follicle of the ovary (forming the inner layer of the VM), the luminal epithelium at the infundibulum of the oviduct (forming the middle and outer layers of the VM), the cultured granulosa cells, and the cells on the VM. The morphological observations were in accordance with previous studies^13, 14, 23^. Innate immune functions in hen reproductive organs) supported the fact that the maternal cells on the VM were mainly granulosa cells. On the other hand, we also attempted to culture the cells on the VM and the granulosa cells of the small yellow follicle (6–8 mm in diameter) using the referencing method^23^. However, the cells on the VM had undergone apoptosis, which led the culture into failure, while the granulosa cells of the SYF could be cultured. The karyotype of the granulosa cells was in agreement with that observed by K. Ladjali-Mohammedi, who reported 78 chromosomes in the chicken, and developed a karyotype for the eight largest chromosomes and the Z and W chromosomes of the female birds^24^.

When the Hoechst 33342 was used to stain the VMs of the unfertilized and fertilized eggs in this study, the maternal cells fluoresced as brightly as the sperm on the VMs. In the research conducted by Arnold, it was found that the fertile eggs were regularly assigned the incorrect sex with the VM samples, while the unfertilized eggs were consistently identified as female. Therefore, it was concluded that the VM was a considerable source of maternal contamination^11^. The present study confirmed that the maternal cells were unevenly distributed, with a constant number at different storage times. Although the specific structural features of VMO I can contribute to its antimicrobial potential, it is generally known that VM quality can be affected by environmental conditions, such as temperature and storage times^4, 25^. The strength of the VM has been found to decrease during prolonged cold storage^26^. This study’s results renewed the knowledge regarding the VM. The live/dead staining of the maternal cells on the VM by the LSCM showed that the cell viability changed with the freshness of the eggs. The maternal cells were found to gradually undergo apoptosis and become degraded during the present study.

The chicken had 2.5 pg DNA per diploid nucleus^27^, so there were more than 3.2 × 105 somatic cells on each vitelline membrane of chicken egg. Sufficient amounts of DNA from the VM were extracted in this study using a standard phenol-chloroform protocol, which could then be amplified for the molecular traceability by microsatellite genotyping. The blood samples were the preferred source of DNA, due to the abundant DNA they contained. Moreover, there were several other destructive and/or nondestructive choices available, such as plucked feathers, buccal swabs, and eggshell swabs^8, 28–32^. However, all of these methods may have disturbed the birds, or the amounts of DNA may have been limited and/or cross-contaminated by other birds. Therefore, the results of this study could potentially supplement the methods which are currently used in molecular genetics.

On the other hand, the DNA samples obtained from one VM of a fresh chicken egg were sufficient for the amplification of 10 microsatellite markers. The WLA-VM was easily distinguished from the WLB-B and RIR-B, while the genotypes of the WLA-VM corresponded to those of the WLA-B. Kazuhiro reported a simple and efficient method for the extraction of PCR-amplifiable DNA from chicken eggshells^33^. This technique was further used to discriminate the eggs of Hinai-jidori from those of other chickens. Therefore, based on the above results, it could be predicted that the VM DNA could be utilized to validate the labeling of specific eggs in the market.

The DNA samples of the quail egg, which represented the smaller eggs (~10 g), were analyzed at different storage times in the present study. In most cases, a small egg means a proportionately smaller yolk^34^. The weight of the quail egg was one fifth that of the chicken egg, and the proportion of the quail yolk was about 35%. However, there were adequate amounts of DNA from the quail VM, 530 ng/VM, for further genetic analysis, which means that the present method can also be applied to the smaller eggs laid by other small birds. On the other hand, it should also be noted that the quail VM became increasingly fragile with extended storage time and was difficult to separate after 14 days of storage at room temperature. Mikail Baylan found significant increases in the pH of albumen with extended storage time^35^. The raised pH caused small abrasions in the VM^34^.

In summary, our study renewed the knowledge of the VM, and illustrated the apoptosis of the granulosa cells on VM with the storage time. The nuclear DNA could be easily extracted using a conventional phenol-chloroform method. The amount of VM DNA allowed researchers to do the further molecular genetic analysis. This method was complementary to the present avian sample techniques due to its easy accessibility, relative stability, and near absence of cross-contamination.

## Methods

### Ethics statement

The animal experiments were approved by the Animal Care and Use Committee of China Agricultural University, and the experiment was performed according to the regulations and guidelines established by this committee.

### Poultry eggs

In this study, the White Leghorn chicken eggs were obtained from the Experimental Unit for Poultry Genetic Resource and Breeding at China Agricultural University. Laying quail eggs were obtained from Hubei Shendan Healthy Food Co., Ltd.

### Isolation of the VM for the microscope examination

All the samples for the VM analysis were collected from White Leghorn chickens in the experimental station of the China Agricultural University. The eggs were broken, and the yolks were separated from the egg whites. A filter paper disc measuring approximately 2 cm in diameter with a hole in the center was placed on the VM to avoid blastodiscs or blastoderm. The filter paper was cut along the outline of the disc, which was then carefully taken off the VM and washed in a petri dish using a phosphate buffered saline (PBS) solution^36^.

### Transmission electron microscopy (TEM)

The VMs of the infertile eggs were fixed in 2.5% glutaraldehyde at 4 °C for three hours, and in 1% osmium tetroxide for another two hours. Then, the specimens were rinsed three times with PBS, each time for five minutes. The membrane were dehydrated with a series of acetone (30%, 50%, 70%, 80%, and 100%), embedded in epoxy resin, and sectioned with an ultramicrotome. The specimens were stained with 50% uranyl acetate in methanol for five minutes, and then in 0.4% lead citrate in saturated NaOH for 10 minutes^37^. The structure of the VM was then examined by TEM, using a JEOL JEM-1230 (JEOL Ltd., Musashino, Akishima, Tokyo, Japan).

### Laser scanning confocal microscopy (LSCM) using a Hoechst 33342 DNA stain

For the Olympus Fluoview^TM^ FV1000 confocal laser microscope scanning, a Hoechst 33342 DNA stain (Sigma-Aldrich, St. Louis, MO, USA; Sigma) was first used to stain the VMs of the fertile eggs and infertile eggs. The VMs were carefully transferred to 24-well plates, which contained 450 μL of PBS in each well, with final concentrations of 5 μM Hoechst 33342^36^. The membrane were incubated for 30 minutes at room temperature under dark conditions, and observed under a fluorescent microscope using the appropriate filters for the Hoechst 33342 excitation and emission. The results obtained the recognitions of the presence of cells with brightly fluorescing nuclei.

### LSCM using Calcein AM/PI

Calcein AM/PI was used to stain the VMs of the eggs at different storage times in order to identify the live cells (green) and dead cells (red)^38^. 40 fresh unfertilized eggs were randomly divided into four groups, with 10 eggs per group. These were then stored at 22 °C (with approximately 50% humidity) for 1, 7, 14, and 21 days, respectively. The VMs were gently separated and transferred to 24-well plates, which contained 300 μL of staining buffer in each well, and 5 μM Live-Dye and 5 μM PI (Live-Dead cell staining kit, BioVision, Milpitas, CA, USA). The VM (2 cm × 2 cm) was incubated for 15 minutes at a temperature of 37 °C under dark conditions, and observed under an LSCM with band-pass filters. The numbers of live and dead cells per 0.4 mm^2^ at the different storage times were analyzed using a one-way ANOVA with IBM SPSS 21. Then, in accordance with the research conducted by Aktan, the surface area of the yolk (approximately 15 cm^2^), and the total numbers of live and dead cells of the entire VM were estimated^39^.

### Histology analysis using HE and Hoechst 33342

The ovary and oviducts were removed from the laying hens. Then, small yellow follicle (SYF, Φ 6–8 mm) on ovary, infundibulum of the oviduct were fixed in with 4% neutral formalin at room temperature for 48 h. Serial tissue sections were cut to 5-μm thickness after embedding in paraffin^14^. The samples of cultured granulosa cells and VM were treated with the method in LSCM part. For optical microscope examination using HE staining, each slide was stained with hematoxylin and eosin (H&E) and then examined by light microscopy (Olympus BX41, Olympus Optical Co., Tokyo, Japan). And for fluorescence observation, dewaxed slides were stained with Hoechst 33342 and examined by Olympus FluoviewTM FV1000 confocal laser microscope.

### Cultivation of chicken granulosa cells

The chickens were euthanized by cervical bleeding post-anesthesia. A pool of SYF (Φ 6–8 mm) was sampled, and the granulosa layers were treated using the method described by Yanmei Jin^23^. Granulosa small sheets were incubated in phosphate-buffered saline (Ca^2+^ and Mg^2+^ free) solution containing 0.2% collagenase (GIBCO BRL). After 20 min of enzyme dissociation at 37 °C, the dispersed cell aggregates were collected and washed twice with a fresh medium. The cell number was counted with a hemocytometer. The cell viability was always over 90%, as determined by the trypan blue exclusion test. The cell suspension was seeded in collagen-treated 96-well culture plates, at a density of 5 × 10^4^/well in 200 μl medium supplemented with 0.5% fetal calf serum (FCS, GIBCO BRL). The cells were incubated at 39 °C in a water-saturated atmosphere of 95% air and 5% CO_2_.

### Chromosome karyotype analysis of granulosa cells

Chicken metaphase chromosome spreads were prepared from 48 h old granulosa cells after treatment with 0.01% colchicine solution. The cells were swollen by treatment with hypotonic KCl, fixed in 1:3 acetic acid/methanol, and dropped onto ethanol cleaned slides. The slides were allowed to air dry and were stored at ± 20 °C. The chromosomes were stained by immersing the slides for 8–10 min in a 10% Giemsa solution^23^. The chromosome images were captured on a Nikon E800 microscope fitted with a cooled CCD (Penguin 150CL, Pixera). These metaphase images were then analysed with CCD and Video Test Karyo version 3.1 software.

### Egg quality measurements

In this research study, 120 unfertilized White Leghorn chicken eggs were randomly divided into four groups, with 30 eggs per group. These were then stored at 22 °C (with approximately 50% humidity) for 1, 7, 14, and 21 days, respectively. The weights of the eggs (EW) and the Haugh unit (HU) were measured using an Egg Multi Tester EMT-5200. Then, the yolks were individually separated and washed with PBS with three repetitions. Each VM was stored in a 1.5 mL tube. The DNA of the VM was extracted using a standard phenol-chloroform extraction^40^. The DNA was re-suspended in 10 μL of TE, and the concentrations were measured using a NanoDrop ND-2000 spectrophotometer (Thermo Scientific, Wilmington, DE, USA). Meanwhile, 120 quail eggs were also randomly divided into four groups, and the egg weight and DNA concentration were determined as described above. At this point, the data were analyzed using a one-way ANOVA with IBM SPSS Version 21.

### Microsatellite genotyping for molecular traceability

The VM of the unfertilized eggs from the White Leghorn A (WLA-VM, n = 30), and the blood samples of the White Leghorn stock A (WLA-B, n = 30) and Rhode Island Red stock (RIR-B, n = 30) were randomly collected from the poultry experimental station of the China Agricultural University. The blood samples of the White Leghorn stock B (WLB, n = 30) were collected from the poultry performance and quality testing center in Beijing.

Then, 10 microsatellite markers were genotyped^41, 42^. These microsatellite markers were found to have high polymorphisms, with the highest number of alleles. Moreover, these markers were distributed on different linkage groups. A PCR was performed in 20 μL of reaction system with DNA, Taq DNA polymerase, PCR buffer with MgCl_2_, and primers. Then, the reaction was incubated at 94 °C for five minutes, with 35 cycles of 94 °C for 30 seconds, 60 to 65 °C (Table 3) for 30 seconds, and 72 °C for 45 seconds, followed by a final extension at 72 °C for 10 minutes. The PCR products were run in a Perkin-Elmer ABI377 automated sequencer (Applied Biosystems, Foster City, CA, USA; AB applied biosystems), and the sizes of the fragments were analyzed using GeneScan 2.0 (AB applied biosystems).

**Table 3:** Primers, PCR conditions, and allele sizes of each microsatellite.

Loci	Primer sequences	Chromosome	Annealing temperature (°C)	Allele size (bp)
ADL176	TTCTCCCGTAACACTCGTCA	2	65.0	192
	TTGTTGATTCTGGTGGTAGC			
ADL181	CAATCTTTTGTGGGGTATGG	2	64.1	178(181)
	CCAGTGAAATTCATCCTTTT			
ADL0278	TGTCATCCAAGAACAGTGTG	8	62.0	100
	CCAGCAGTCTACCTTCCTAT			
LEI094	CACAGTGCAGAGTGGTGCGA	4	65.0	188
	CAGGATGGCTGTTATGCTTCCA			
LEI228	AGCGTACCTGATAATGATGAGC	2	64.1	214
	GCTGGGTTATTTCAATATGTGG			
LEI0166	TATCCCCTGGCTGGGAGTT	3	60.0	360
	CTCCTGCCCTTAGCTACGCA			
LEI0258	AGCTGTGCTCAGTCCTCAGTGC	16	64.5	300
	CACGCAGCAGAACTTGGTAAGG			
MCW67	GAGATGTAGTTGCCACATTCCGAC	10	62.5	180
	GCACTACTGTGCTGCAGTTT			
MCW183	TGAGATTTACTGGAGCCTGCC	7	64.5	290–311
	ATCCCAGTGTCGAGTATCCGA			
MCW0330	AATGTTCTCATAGAGTTCCTGC	17	62.5	256–300
	TGGACCTCATCAGTCTGACAG			

The Nei standard genetic distances were estimated using a DISPAN package^43^. A factor correspondence analysis was performed using the program GENETIX 4.05 in order to detect the population structure based on the allele frequencies.
